# Individual-level changes in self-rated health before and during the economic crisis in Europe

**DOI:** 10.1186/s12939-015-0290-8

**Published:** 2016-01-05

**Authors:** Dawit Shawel Abebe, Anne Grete Tøge, Espen Dahl

**Affiliations:** NOVA, Oslo and Akershus University College, P.O. Box: 4, St. Olavs plass, Oslo, NO-0130 Norway; Faculty of Social Sciences, Oslo and Akershus University College, Oslo, Norway

**Keywords:** Self-rated health, EU-SILC, Health inequality, Trends

## Abstract

**Background:**

Changes over time in self-rated health (SRH) are increasingly documented during the current economic crisis, though whether these are due to selection, causation, or methodological artefacts is unclear. This study accordingly investigates changes in SRH, and social inequalities in these changes, before and during the economic crisis in 23 European countries.

**Methods:**

We used balanced panel data, 2005–2011, from the European Union Statistics on Income and Living Conditions (EU-SILC). We included the working-age population (25–60 years old) living in 23 European countries. The data cover 65,618 respondents, 2005–2007 (pre-recession cohort), and 43,188 respondents, 2008–2011 (recession cohort). The data analyses used mixed-effects ordinal logistic regression models considering the degree of recession (i.e., pre, mild, and severe).

**Results:**

Individual-level changes in SRH over time indicted a stable trend during the pre-recession period, while a significant increasing trend in fair and poor SRH was found in the mild- and severe-recession cohorts. Micro-level demographic and socio-economic status (SES) factors (i.e., age, gender, education, and transitions to employment/unemployment), and macro-level factors such as welfare generosity are significantly associated with SRH trends across the degrees of recession.

**Conclusions:**

The current economic crisis accounts for an increasing trend in fair and poor SRH among the general working-age population of Europe. Despite the general SES inequalities in SRH, the health of vulnerable groups has been affected the same way before and during the current recession.

**Electronic supplementary material:**

The online version of this article (doi:10.1186/s12939-015-0290-8) contains supplementary material, which is available to authorized users.

## Background

The impact of economic crisis on health is a global concern, particularly among vulnerable groups, such as youth, recent immigrants, single mothers, the less educated, and low-income households, as economic crisis could widen pre-existing inequalities in health [1, 2]. However, research provides little insight into changing health trends at the individual level and therefore limited evidence for casual mechanisms.

In general, individual vulnerability can be derived from two types of mechanisms, coping and social stress. Coping mechanisms are individual processes, though they are influenced by the social environment. Witnessing how peers handle challenges both affects the perceived “normality” of given problems and provides information on successful ways of coping with them. If coping mechanisms are prevalent, one should expect decreasing negative effects of recessions as a larger share of the population is affected by their consequences [3–5]. Social stress theories postulate that individual stress is mitigated by personal, material, and social resources. The amount of transfer of such resources reduces the probability of risk factors becoming actual vulnerability [6]. During an economic crisis, the restricted availability of economic resources could limit people’s abilities (particularly among those already susceptible) to cope with both their own situation and interpersonal relationships [6]. There is no reason to believe that coping and social stress mechanisms vary between countries, however, these mechanisms might be important in explaining how changes in environment (that vary across countries) affect individuals.

An additional factor in this situation is the impact of welfare state systems and qualities [7], as it is not necessarily the actual economic crisis but rather the policy responses to it that determine the health impact [8–10]. The financial collapse and economic stagnation did not translate into adverse health outcomes in Iceland, a country that refused to bail out banks and implement austerity policies, while health changes are documented in countries that introduced austerity, such as Greece, Spain, and Portugal [11].

Regarding health inequalities, the research is inconsistent. For instance, findings from Greece, Lithuania, Poland, and Estonia indicate increased proportions of individuals with poor self-rated health (SRH) during the economic crisis [12–15], particularly among the unemployed [16], the elderly, and less-educated women [13]. However, a stable proportion of individuals with poor or even declining SRH was found among the general population in Finland [13] and Spain [16], respectively. Although income-related health inequalities were documented in Iceland, changes in SRH were found to be stable before and after Iceland’s economic collapse [17]. As none of these studies examined individual-level changes in SRH across degrees of recession, they provide limited evidence regarding the causal effect of the crisis.

Most prior studies used a repeated cross-sectional design to compare changes in health outcomes before and after the economic crisis. Such designs are likely biased due to omitted time-variant variables [18], particularly changes in sample composition, which introduce uncertainty in determining a causal pathway from crisis and policy responses to health changes. Another challenge is short follow-up periods, which could mask outcome changes over time. Examining individual health changes using a long-term longitudinal design is recommended as it provides estimates closer to the causal effects. Such a design is also useful for subgroup analyses, as it allows trends in different social groups to be investigated [19–21, 13].

The current study examines changes in SRH before and during the economic crisis and how micro- and macro-level socio-economic status (SES) indicators relate to changes in SRH before and during the crisis in 23 European countries. The study specifically aimed to investigate trends and predictors of SRH across the severity of recessions – pre-, mild- and severe recessions – among the general working-age population in Europe. Exploring changes in SRH before and during the economic crisis may provide important indications about the effects of economic crisis on health and health inequalities, which have important implications for the development of interventions to reduce social inequalities in health.

## Methods

### Participants

The data were extracted from two panels of the European Union Statistics on Income and Living Conditions (EU-SILC) from 2005 to 2011: 2005–2007 constitutes the pre-recession cohort and 2008–2011 the recession cohort. A balanced panel data structure was used. The sample population was further limited to the working-age population (25–60 years old) living in one of the 23 countries that participated in both periods.^1^ The net sample included 65,618 respondents in 2005–2007 and 43,188 respondents in 2008–2011. The study and country-specific sampling procedure are thoroughly documented in MISSY – Metadata for Official Statistics.

During the recession period (2008–2011), we classified participants into mild- and severe-recession cohorts using changes in the median unemployment rates of countries between the pre- and during-crisis periods. Countries with a ≥1 percentage point increase in median unemployment during the crisis were regarded as experiencing severe recession, while those with a <1 percentage point increase were categorized as experiencing mild recession (see note in Tables 3 and 4 for the list of countries). This cut-off point corresponds to the median change in unemployment between the pre- and during-crisis periods in 23 European countries, i.e., 1.1 percentage points. Although GDP change is usually used to define recessions [22], change in unemployment is considered a better proxy for the social impact of recessions than is GDP growth because countries may experience “jobless growth,” for example.

### Dependent and independent variables

#### Outcome

Mean scores for self-rated health

SRH was measured using a single self-rated item, “How is your health in general?” Answers were ranked on a five-point scale, i.e., 5 = “very good”, 4 = “good”, 3 = “fair”, 2 = “bad”, and 1 = “very bad”. Although this item is commonly used as a dummy variable, we opted to conduct the analyses using SRH as an ordinal variable. As ordinal categories could be unevenly spaced, i.e., the gap between those reporting “very good” and “good” could be small, while the gap between “good” and “fair” may be large [23], we thus categorized SRH into three levels, such that 0 = “very good or good”, 1 = “fair”, and 2 = “bad or very bad”/ “poor”.

### Predictors and control variables

Age was categorized into two groups: 0 = 25–40 and 1 = 40–60 years old. Male was coded as 0 and female as 1.

Education was measured according to the International Standard Classification of Education (ISCED), and was coded as 0 for those with secondary or lower education and 1 for those with tertiary education.

Unemployment was coded according to the self-reported status at the time of the interview: 1 = unemployed and 0 = employed.^2^ Following the Mundlak approach [24], this variable was recoded into a variable denoting the within-individual mean (across time) and a variable denoting the time-specific deviation from this mean. The time-variant variable was then separated into two transitions: from employment to unemployment (“unemployment transition”) and from unemployment to employment (“employment transition”).

Welfare generosity, unemployment rates, and Gini coefficients were included as country-level variables. Welfare generosity refers to the yearly sum of social expenditure (purchasing power standard) per inhabitant on family/children, unemployment, sickness/healthcare/disability, and housing and social exclusion benefits, as there is more variation in the overall generosity than in how the spending is prioritised (see Additional file1). This sum is divided by the inverse of the employment rate among those 20–64 years old [25]. We used the average welfare generosity scores in 2004 and 2006 for the pre-crisis period and the average scores in 2008 and 2010 for the during-crisis period. Unemployment rates (in percent among those 25–74 years old), Gini coefficients, and GDP growth rates per year (2005–2011) per country were imported from the Eurostat database.

In addition, the following micro-level variables were included as covariates: baseline SRH, baseline employment status, marital status, and household income.

### Statistical analysis

Mixed-effects ordinal logistic regression models were employed to investigate the individual-level changes and predictors of the SRH status over time (i.e., the 2005–2007 pre-crisis period vs. the 2008–2011 during-crisis period). These models are recommended for modelling individual trajectories over time in longitudinal studies, and have the advantage of controlling for dependence among the repeated responses of a subject [26, 27]. The following mathematical equation represents the basic model:

$$ \mathbf{\mathsf{y}}\mathbf{\mathsf{i}}\mathbf{\mathsf{j}}=\boldsymbol{\upbeta} \mathbf{\mathsf{1}}+\boldsymbol{\upbeta} \mathbf{\mathsf{2}}\boldsymbol{\upchi} \mathbf{\mathsf{2}}\mathbf{\mathsf{j}}+\cdot \cdot \cdot +\boldsymbol{\upbeta} \mathbf{\mathsf{3}}\boldsymbol{\upchi} \mathbf{\mathsf{3}}\mathbf{\mathsf{i}}\mathbf{\mathsf{j}}+\cdot \cdot \cdot +\boldsymbol{\upzeta} \mathbf{\mathsf{1}}\mathbf{\mathsf{j}}+\boldsymbol{\upzeta} \mathbf{\mathsf{2}}\mathbf{\mathsf{j}}\boldsymbol{\upchi} \mathbf{\mathsf{i}}\mathbf{\mathsf{j}}+\boldsymbol{\upvarepsilon} \mathbf{\mathsf{i}}\mathbf{\mathsf{j}} $$

where *y* = outcome (SRH categories), *χ* = covariate (predictor), *i* = time point (occasion), *j* = subject, and *ε*_*ij*_ = residuals that are independent across subjects and occasions. The model has two parts, fixed and random effects. A fixed effect represents a single value, β, existing in the population and assumed to be shared by all individuals: β_1_ = the intercept (i.e., starting point) and β_2j_ = the regression coefficient (i.e., mean slope) of time-invariant predictors (e.g., gender), while β_3ij_ = the regression coefficient of time-variant predictors (e.g., unemployment transition). For a linear trajectory, these estimates of the mean intercepts and slopes jointly define the underlying trajectory pooling of the entire sample. The random effects are estimates of the between-person variability in the individual intercepts and slopes. They describe subject-specific characteristics, i.e., ζ_1j_ and ζ_2j_ represent the random intercept and random slope in the basic equation, respectively.

For the purpose of study, the fixed effects (β) are presented and discussed. Since log odds ratios in ordinal logistic regression are not comparable across models due to unobserved heterogeneity and difficult to interpret because they are relative to the base outcome (i.e., very good/good SRH), results from multinomial ordinal regression models are presented as Average marginal effects (AME). AME eases the interpretation of results since they report the averaged change in probability (P(y = 1)) given the distribution of other independent variables for all observations. For all analyses, a *p*-value under 0.05 was considered statistically significant. Statistical analysis was conducted using Stata SE/13 for Windows.

## Results

A descriptive summary of all variables and covariates over time is displayed in Table 1. Country-specific ordinal logistic regression models were first constructed to describe changes in the SRH status before and during the crisis period. As shown in Table 2, unadjusted regression estimates and standard errors are presented for each country (i.e., describing changes in the SRH status over time). In the pre-recession period, individuals in most countries had a stable SRH trend (*N* = 14, 60.8 %) or a declining trend in fair or poor SRH status (*N* = 6, 26.1 %), except individuals in Spain, Hungary and Netherland, who had significantly an increasing trend in fair or poor SRH over time. Individuals in elven countries (47.8 %) had stable or decreasing trends in fair or poor SRH before the crisis, but increasing in fair or poor SRH during the crisis. Still, individuals in eleven countries (47.8 %) maintained stable SRH during the crisis. Exceptionally, individuals in Spain displayed a declining trend in fair or poor SRH during the crisis.

**Table 1:** Descriptive summary of study participants (balanced panel)

Variables	Pre-crisis period			During-crisis period			
	2005	2006	2007	2008	2009	2010	2011
SRH, N (%)							
ᅟVery good/good	35,715 (65.8)	36,145 (66.6)	35,489 (65.1)	25,626 (70.5)	25,307 (70.9)	25,467 (70.8)	25,056 (70.7)
ᅟFair	13,641 (25.1)	13,514 (24.9)	12,264 (23.5)	8039 (22.1)	7873 (22.1)	7983 (22.2)	7722 (21.8)
ᅟBad/very bad	4916 (9.1)	4642 (8.7)	4396 (8.4)	2668 (7.4)	2499 (7.0)	2518 (7.0)	2638 (7.5)
Age (years), N (%):							
ᅟ25–40	27,169 (41.7)	26,921 (41.0)	25,415 (40.2)	16,900 (39.1)	16,372 (38.5)	16,134 (37.5)	15,642 (36.8)
ᅟ40–60	38,014 (58.3)	38,697 (59.0)	37,766 (59.8)	26,288 (60.9)	26,267 (62.5)	26,897 (62.5)	26,821 (63.2)
Education, N (%):							
ᅟLess than tertiary	50,902 (78.1)	50,939 (77.6)	48,601 (76.9)	31,971 (74.0)	31,335 (73.5)	31,301 (72.7)	30,485 (71.8)
ᅟTertiary	14,281 (21.9)	14,679 (22.4)	14,580 (23.1)	11,217 (26.0)	11,304 (26.5)	11,730 (27.3)	11,978 (28.2)
Employment status, N (%):							
ᅟEmployed	46,065 (71.2)	47,330 (72.9)	46,291 (74.2)	32,495 (75.9)	32,672 (74.8)	31,868 (74.6)	31,630 (75.0)
ᅟUnemployed	4808 (7.4)	4349 (6.7)	3685 (5.9)	2241 (5.2)	2950 (6.9)	3335 (7.8)	3359 (7.9)
ᅟOther	13,834 (21.4)	13,246 (20.4)	12,397 (19.9)	8061 (18.9)	7731 (18.3)	7526 (17.6)	7162 (16.9)
ᅟGDP, M (SD)	3.44 (2.01)	4.76 (2.09)	4.82 (2.72)	0.97 (2.14)	−4.83 (4.00)	1.79 (1.73)	2.12 (2.06)
ᅟGini, M (SD)	29.78 (4.41)	29.06 (4.07)	28.87 (3.73)	28.90 (3.69)	29.01 (3.97)	28.94 (3.96)	29.03 (3.75)
ᅟWelfare generosity, M (SD)	144.08 (98.31)	141.88 (97.33)	139.26 (96.25)	186.81 (118.01)	184.93 (117.93)	180.74 (117.47)	180.40 (117.39)
ᅟUnemployment rate per country, M (SD)	8.68 (4.22)	7.61 (3.13)	6.31 (2.14)	6.28 (2.31)	8.89 (4.07)	10.17 (4.77)	9.89 (4.66)

*M* mean, *SD* standard deviation, *N* number, *SRH* self-rated health (higher mean score indicates better SRH), and *GDP* gross domestic product growth rate

**Table 2:** Fixed effect estimates from multinomial ordinal logistic regression models describing individual-level changes in SRH over time before and during the economic crisis across 23 countries

	Pre-crisis period			During–crisis period		
Country	β	SE	Change^a^	β	SE	Change^a^
Austria	−0.025	0.055	S	0.091	0.040	I
Belgium	0.063	0.055	S	−0.041	0.047	S
Cyprus	−0.034	0.052	S	0.036	0.050	S
Czech Republic	−0.104	0.034	S	<-0.001	0.037	S
Denmark	0.082	0.095	S	0.132	0.060	I
Estonia	0.110	0.072	S	0.118	0.038	I
Spain	0.132	0.032	I	−0.065	0.025	D
Finland	−0.162	0.069	D	0.018	0.053	S
France	−0.069	0.098	S	0.162	0.025	I
Hungary	0.111	0.028	I	0.059	0.028	I
Iceland	0.319	0.165	S	0.116	0.107	S
Italy	−0.165	0.025	D	−0.037	0.020	S
Lithuania	−0.172	0.049	D	0.229	0.046	I
Luxemburg	−0.032	0.186	S	0.198	0.054	I
Latvia	−0.275	0.040	D	0.102	0.029	I
Netherlands	0.142	0.045	I	0.088	0.048	S
Norway	0.467	0.265	S	0.129	0.047	I
Poland	−0.106	0.020	D	−0.026	0.022	S
Portugal	−0.055	0.075	S	0.063	0.038	S
Sweden	−0.119	0.084	S	−0.031	0.063	S
Slovenia	−0.075	0.040	S	0.067	0.036	S
Slovakia	−0.079	0.049	S	0.085	0.040	I
UK	−0.055	0.041	S	0.170	0.046	I

*β* regression coefficients, *SE* standard error, *SRH* self-rated health

β represents regression coefficients measuring the probability of change towards fair or poor SRH status over time (i.e., very good/good SRH was a reference category)

^a^Indicates the individual patterns of change in the status of SRH over time: S = stable in SRH status, D = significant decline in fair or poor SRH status and I = significant increase in fair or poor SRH status

To further examine the SRH trajectories and predictors, multivariate ordinal logistic regression models were applied according to the severity of recessions (pre-, mild- and severe-recession cohorts). Tables 3 and 4 present AME (standard error in parentheses) results for the fair and poor SRH status, respectively. In Model 1, first, we described changes in SRH over time across the degrees of recession. In Model 2, we then added micro- and macro-level factors to estimate how they independently predict changes in SRH among all individuals in the pre-, mild-, and severe-recession cohorts over time.

**Table 3:** Average marginal effects from multivariate multinomial ordinal models (fixed effects) showing micro- and macro-level predictors of fair SRH over time among countries during pre-, mild-, or severe-recession

	Pre-recession	Mild recession	Severe recession
Predictors	AME (SE)	AME (SE)	AME (SE)
Model 1			
Time (years)	−0.005 (0.001)***	0.003 (<0.001)***	0.005 (<0.001)***
Model 2			
Individual-level:			
Time (years)	−0.008 (0.001)***	−0.003 (<0.001)**	0.001 (0.001)
Gender (male vs. female)	0.014 (0.002)***	0.002 (0.003)	0.012 (0.002)***
Age (25–40 vs. 40–60 years)	0.116 (0.002)***	0.101 (0.004)***	0.078 (0.003)***
Tertiary education	−0.089 (0.002)***	−0.071 (0.004)***	−0.060 (0.003)***
Transition to employment	−0.062 (0.004)***	−0.097 (0.011)***	−0.038 (0.008)***
Transition to unemployment	0.074 (0.008)***	0.041 (0.012)**	0.039 (0.008)***
Country-level:			
Welfare generosity	−0.105 (0.002)***	−0.039 (0.004)***	−0.082 (0.004)***
Gini	0.077 (0.008)***	0.014 (0.019)	0.071 (0.012)***
Observations (person-years)	159,303	47,157	34,840
Number of participants	58,605	16,537	19,197
Number of countries	23	12	11

AME indicates the averaged change in probability (P(y = 1)) given the distribution of other independent variables for all observations. AME controlled for baseline employment status, marital status, household income, and GDP growth rate

Welfare generosity and Gini coefficients were transformed into natural logarithms

Transition variables had values ranging from 0 to 1, where 0 indicates “always employed” and 1 indicates “always unemployed” during the study period

Mild-recession countries were Austria, Belgium, Czech Republic, Finland, France, Luxembourg, the Netherlands, Norway, Poland, Slovakia, Slovenia, and Sweden

Severe-recession countries were Cyprus, Denmark, Estonia, Hungary, Iceland, Italy, Latvia, Lithuania, Portugal, Spain, and the UK

*** *p* < 0.001, ** *p* < 0.01, * *p* < 0.05; AME = average marginal effects; SE = standard error

**Table 4:** Average marginal effects from multivariate multinomial ordinal models (fixed effects) showing micro- and macro-level predictors of poor SRH over time among countries during pre-, mild-, or severe-recession

	Pre-recession	Mild recession	Severe recession
Predictors	AME (SE)	AME (SE)	AME (SE)
Model 1			
Time (years)	<-0.001 (<0.001)***	0.001 (<0.001)***	0.001 (<0.001)***
Model 2			
Individual-level:			
Time (years)	−0.003 (<0.001)***	−0.001 (<0.001)**	0.001 (0.001)
Gender (male vs. female)	0.006 (0.001)***	<0.001 (0.001)	0.006 (0.001)***
Age (25–40 vs. 40–60 years)	0.045 (0.001)***	0.033 (0.002)***	0.043 (0.002)***
Tertiary education	−0.034 (0.001)***	−0.023 (0.001)***	−0.033 (0.002)***
Transition to employment	−0.024 (0.002)***	−0.033 (0.001)***	−0.022 (0.004)***
Transition to unemployment	0.028 (0.003)***	0.013 (0.003)***	0.022 (0.004)***
Country-level:			
Welfare generosity	−0.041 (0.001)***	−0.013 (0.001)***	−0.045 (0.002)***
Gini	0.029 (0.003)***	0.005 (0.006)	0.039 (0.006)***
Observations (person-years)	159,303	47,157	34,840
Number of participants	58,605	16,537	19,197
Number of countries	23	12	11

AME indicates the averaged change in probability (P(y = 1)) given the distribution of other independent variables for all observations. AME controlled for baseline employment status, marital status, household income, and GDP growth rate

Welfare generosity and Gini coefficients were transformed into natural logarithms

Transition variables had values ranging from 0 to 1, where 0 indicates “always employed” and 1 indicates “always unemployed” during the study period

Mild-recession countries were Austria, Belgium, Czech Republic, Finland, France, Luxembourg, the Netherlands, Norway, Poland, Slovakia, Slovenia, and Sweden

Severe-recession countries were Cyprus, Denmark, Estonia, Hungary, Iceland, Italy, Latvia, Lithuania, Portugal, Spain, and the UK

*** *p* < 0.001, ** *p* < 0.01, * *p* < 0.05; AME = average marginal effects; SE = standard error

Results in Model 1 in the Tables 3 and 4 showed that significant declining trends in fair and poor SRH before the crisis, while increasing trends in the mild and severe recession cohorts. Multivariate results in Model 2 in the Tables 3 and 4 indicate that women had greater risk to experience fair and poor SRH than males in pre- and severe-recession cohort. The older age group displayed a more significant risk to fair and poor SRH than did the younger age group regardless of the degree of recession. Having tertiary education, transition to employment and living in more-welfare-generous countries were significantly associated with a lower risk to fair and poor SRH in all cohorts over time. Transition to unemployment was significantly positively related to fair and poor SRH regardless of the degree of recession. Living in a country with a higher Gini coefficient significantly predicted fair and poor SRH among individuals in the pre- and severe-recession cohorts.

Although univariate regression analyses showed that gender (β = 0.34, *p* < 0.001) and Gini coefficient (β =2.69, *p* < 0.001) significantly associated with SRH over time in the mild recession cohort, these associations did not retain statistical significance in multivariate regression, which could be due to multicollinearity. Furthermore, the adjusted estimates of “time” in Model 2 in the Tables 3 and 4 revealed that trends in fair and poor SRH appear to decline with time in the mild recession cohort, while showed a stable trend in the severe recession cohort.

## Discussion

This study found that the working-age population in European countries in general experienced an increasing trend in fair and poor SRH during the current crisis regardless of the severity of recessions. These changes in SRH during the crisis periods became stable or even declined in the fair and poor SRH status when adjusted to micro- and macro-levels predictors. This suggests that micro- and macro-levels predictors such as age, gender, levels of education, employment status, welfare generosity and Gini coefficients, could account for the SRH trends during the recession periods. However, the country-specific trends for the changes of SRH during the crisis period revealed mixed findings; about half of the countries studied had a stable SRH trend during the crisis, while the rest half showed an increasing trend in fair and poor SRH, except individuals in Spain – experiencing a declining trend in fair and poor SRH during the crisis period. Future research should focus in examining underlying mechanisms explaining such country-specific variations in changes of SRH over time, which may add important insights in a debate about the impact of economic crisis on health and health inequalities.

We found no evidence of elevated health effects among vulnerable groups – low educated, unemployed and living in countries with less welfare generosity and increased inequality, whose SRH does not seem to be more affected by severe than mild or pre- recessions. Similarly, regarding exposure, the health effects of unemployment and employment transitions do not differ significantly across severe-, mild-, and pre-recessions. This could indicate resilience and a substantial prevalence of coping mechanisms among the (assumed) vulnerable groups and individuals, which they may draw from family, social networks, and community resources [28].

In addition, this study identified SES indicators predicting SRH changes across the degrees of recession. For instance, although men have historically been found to be more vulnerable to deteriorating health during economic downturns [28], the present study found that women tended to be more affected than men before the crisis as well as among the severe recession countries, while no gender differences were found among the mild-recession countries. The growing participation of women in the labour market could be one explanation of this [28, 29]. Socio-demographic disparities in SRH also remained the same across the degrees of recession, those who were older and less educated being more prone to SRH deterioration regardless of the degree of recession. Such persistence in socio-demographic inequalities over time is likely rooted in a person’s life course [30], indicating that health among disadvantaged groups may have been affected the same way before and during the current recession.

The analyses also found that unemployment transitions significantly predicted the trend in SRH regardless of the degree of recession. Although the health of unemployed individuals is sometimes expected to decline with high unemployment rates [31], unemployed individuals have also been found displaying similar or even better mental health compared with employed individuals during periods of high unemployment [31–33]. Lower risks of self-blame and social stigmatization at times of high unemployment make it more acceptable to attribute individual unemployment to external causes. Such an increased tendency to externalize the causes of one’s own unemployment may offset the unemployment-related stress stemming from a lower probability of reemployment. Hence, our results support the finding that the impact of transitions to unemployment or employment on SRH may not necessarily increase during recession periods.

The findings further indicated that welfare generosity can buffer the declining trend in SRH regardless of the severity of recessions. The effect of welfare generosity could imply that social stress processes supplement the coping mechanism [34], particularly for mental health among vulnerable groups, as the probability of participation in social networks increases in line with welfare generosity [25]. Additionally, changes in inequality indicated by increased Gini coefficients appear to be significantly related to declining SRH, though these associations are only significant in the pre- and severe-recession cohorts.

This longitudinal study is the first to examine individual SRH trends across degrees of recession using a large sample of individuals representing the working-age population of Europe, which lets us observe current macroeconomic changes and their effect on health. Unlike most prior studies, which find that selection into and out of unemployment moderates health changes [36], the longitudinal design of this study allows a comparative analysis of health changes across time and space, more directly investigating the impact of micro- and macro-level factors.

The study has some limitations warranting consideration. First, SRH is a rough measure of health, where it is impossible to distinguish between mental and physical symptoms. On the other hand, SRH measures self-perceived illness independent of diagnosis (disease) and societal acknowledgement of the health issues (sickness) [35], which means that SRH could be more sensitive to minor changes in health status than diagnosis and less sensitive to attitudes than sick-leave. Nevertheless, the reliability of SRH relies on the assumption that the respondents actually “know” their own health and report correct levels [36]. It is impossible to measure respondent’s self-knowledge in health in the EU-SILC as there are only self-reported measurements. However, a literature review of 27 studies found that SRH represents an independent predictor of health status [37]. Second, unemployment rates were only applied to characterize the degree of recession. Although the unemployment rate is a prime indicator of recession, combining it with other macro-level indicators (e.g., proportion of workless households and real GDP) quantifying austerity and policy responses would provide an index better characterizing the degree of recession. Third, comparisons of trends in a given outcome across cohorts of countries could be broad and heterogenic, possibly creating non-differential misclassification bias resulting in underestimation of the true strength of an association between SRH changes and degree of recession. It also ignores the variations in the timing of economic crisis between countries. Moreover, a country-specific trend could differ from a cohort trend; as demonstrated in our analyses, about half of countries maintained stable SRH during the crisis period. Finally, differences in sample size across countries and over time accompanied by attrition difficulties make the panel sample less representative than it could be. Albeit our estimates are closer to the causal effects than in repeated cross-sectional studies, these limitations suggests that the results should not be interpreted as the true causal effect size.

## Conclusion

This study examines the whole spectrum of SRH changes from pre-recession to mild- and severe-recession conditions among the general working-age population of Europe. Compared with previous research, it provides more accurate conclusions about the casual relationships between the SRH trend and micro- and macro-level indicators across periods of economic up- and downturns. Although micro- and macro-level SES predictors are significantly related to the SRH trend over time, no differences were found in the effects of such predictors across degrees of recession. This may imply that mechanisms underlying health inequalities appear to be similar between pre- and during recession periods, suggesting the persistence of health inequalities over time as well as stronger emphasis on interventions to prevent negative health effects among the vulnerable groups regardless of the severity of recessions.

## Additional file

Additional file 1:
**Levels of social spending for different benefits among European countries in 2010.** (XLS 57 kb)

## Abbreviations

EU-SILC: European union statistics on income and living conditions
ILO: International labor organization
SRH: Self-rated health

## Footnotes

1 The following countries were included: Austria, Belgium, Czech Republic, Cyprus, Denmark, Estonia, Finland, France, Hungary, Iceland, Italy, Latvia, Lithuania, Luxembourg, the Netherlands, Norway, Poland, Portugal, Slovakia, Slovenia, Sweden, Spain, and the UK.

2 The ILO definition of unemployment requires active job seeking. However, as many may have given up and no longer be actively job seeking, this definition is not useful as a measure of unemployment in this study.
