# Erratum to: individual-level changes in self-rated health before and during the economic crisis in Europe

**DOI:** 10.1186/s12939-016-0340-x

**Published:** 2016-03-23

**Authors:** Dawit Shawel Abebe, Anne Grete Tøge, Espen Dahl

**Affiliations:** NOVA, Oslo and Akershus University College, P.O. Box: 4St. Olavs plass, Oslo, NO-0130 Norway; Faculty of Social Sciences, Oslo and Akershus University College, Oslo, Norway

Unfortunately, after publication of this article [1], it was noticed that there errors within the article. In the Background and Conclusion sections, the two uses of the word “casual” was intended to be “causal”. In the Results section, the text “elven countries” should instead read “eleven countries”.
